# Discrete Phase Shifts of Intelligent Reflecting Surface Systems Considering Network Overhead

**DOI:** 10.3390/e24121753

**Published:** 2022-11-30

**Authors:** Jaehong Kim, Heejung Yu, Xin Kang, Jingon Joung

**Affiliations:** 1 School of Electrical and Electronics Engineering, Chung-Ang University, Seoul 06974, Republic of Korea; 2 Department of Electronics and Information Engineering, Korea University, Sejong 30019, Republic of Korea; 3 Center for Intelligent Networking and Communications (CINC), University of Electronic Science and Technology of China (UESTC), Chengdu 611731, China

**Keywords:** intelligent reflecting surface, discrete phase shift, signaling overhead, block coordinate descent, greedy algorithm, incremental search

## Abstract

In this study, the performance of intelligent reflecting surfaces (IRSs) with a discrete phase shift strategy is examined in multiple-antenna systems. Considering the IRS network overhead, the achievable rate model is newly designed to evaluate the practical IRS system performance. Finding the optimal resolution of the IRS discrete phase shifts and a corresponding phase shift vector is an NP-hard combinatorial problem with an extremely large search complexity. Recognizing the performance trade-off between the IRS passive beamforming gain and IRS signaling overheads, the incremental search method is proposed to present the optimal resolution of the IRS discrete phase shift. Moreover, two low-complexity sub-algorithms are suggested to obtain the IRS discrete phase shift vector during the incremental search algorithms. The proposed incremental search-based discrete phase shift method can efficiently obtain the optimal resolution of the IRS discrete phase shift that maximizes the overhead-aware achievable rate. Simulation results show that the discrete phase shift with the incremental search method outperforms the conventional analog phase shift by choosing the optimal resolution of the IRS discrete phase shift. Furthermore, the cumulative distribution function comparison shows the superiority of the proposed method over the entire coverage area. Specifically, it is shown that more than 20% of coverage extension can be accomplished by deploying IRS with the proposed method.

## 1. Introduction

An intelligent reflecting surface (IRS) that can efficiently control the wireless environment with low cost and energy is one of the most promising technologies for future wireless communication systems [1,2,3,4,5,6,7,8,9,10,11,12,13,14,15,16]. The IRS, also known as reconfigurable intelligent surface [17,18,19], large intelligent surface [20,21,22,23], large intelligent metasurface [24], smart reflect-arrays [25,26], software-defined metasurfaces [27,28], passive intelligent surface [29,30], and passive intelligent mirrors [31], is an artificial surface that consists of a large number of passive and low-cost reflecting elements made of meta-materials [18,21,32,33]. By controlling the active elements, such as positive–intrinsic–negative (PIN) diodes and varactors, the resistance and capacitance of each IRS element can be adjusted [33], which enables each element to independently control the phase of the reflected signals. The phase shifts of the incoming signals can steer the direction of the propagation and generate additional wireless links. Thus, by intelligently controlling the phase shifts of IRS elements such that the intended signals are concentrated and/or interferences are mitigated, the wireless link quality can be significantly enhanced. Furthermore, the passive operation of IRS without amplifiers enables the communication systems to efficiently operate energy [17,18,21,32,33]. Therefore, the IRS technique has been vigorously applied to various wireless communication systems, such as the multi-user multiple-input single-output (MISO) systems [5,34,35], secure-communication systems [6,7,8], multi-cell MISO systems [9], broadcasting systems [10,36], multi-group multicast MISO systems [14], non-orthogonal multiple access (NOMA) systems [11,12,13,22], millimeter-wave systems [15,16,37], and cognitive radio or symbiotic radio systems [38,39]. The majority of IRS techniques have mainly been applied to beamforming systems by exploiting the channel state information (CSI) at the transmitter [9,10,11,39]. Using the CSI, the transmitter can design the IRS phase shift vector and beamforming vector of the transmitter, which are called passive and active beamforming design, respectively, [5].

Although the passive beamforming gain of IRS can be maximized by elaborately controlling the phase shift value of each reflecting element in the analog domain, the IRS analog phase shift methods remain two main practical issues: (i) *network overhead, i.e., signal overhead,* [23,40,41] and (ii) *IRS implementation cost* [4,42]. Unless a dedicated (wired or wireless) link is available for the phase shift information sharing between a base station (BS) and the IRS, the wireless communication channels should be shared for the IRS control, which would be a burden on the network. The amount of IRS phase shift information is significantly large, especially for the analog phase shift IRS, because the high-resolution phase control is required to represent the analog phase shift values. Moreover, because the amount of IRS phase shift information is proportional to the number of IRS elements, the network overhead of massive IRS systems may be unaffordable. Furthermore, the analog phase shift IRS is practically difficult to implement due to the hardware complexity and its resulting cost. Specifically, to implement a single IRS reflecting element with *L*-level phase resolution, at least $\log_{2}L$ PIN diodes are required [32], which increases the fabrication cost, the instability of phase states [42], and the power consumption of the total IRS planar [4]. Although a single varactor diode can be employed to represent the analog phase shift value, its requirement of wide-range bias voltage makes it more costly to implement [4]. In [17,43], it was shown that the element-wise IRS power consumption is non-negligible if the number of analog phase-shift elements is enormous. Thus, it is challenging to implement an IRS system with a large number of analog phase shift IRS elements.

Taking the network overhead and IRS implementation cost issues into account, the IRS phase shift methods were considered with a limited phase shift resolution, which is called a *discrete phase shift*. For example, the IRS discrete phase shift methods were utilized in MISO systems [44,45,46,47,48], device-to-device [43,49], Internet of Things [50,51], coordinated multipoint transmission [52], millimeter-wave system [53], NOMA systems [54,55], and orthogonal frequency-division multiplexing (OFDM) systems [56]. Particularly, Ref. [44] evaluated the effect of received power loss with respect to the resolution of IRS discrete phase shift and compared the performance of discrete phase shift to the ideal analog phase shift method. In [46], the performance of channel estimation with the low-resolution discrete phase shift was evaluated. Furthermore, [45] discussed the effect of discrete phase resolution on the data rate in the uplink single antenna systems. However, there have been no attempts to design the optimal IRS discrete phase shift resolution considering the network overhead of IRS-aided multiple-antenna systems.

In this study, focusing on the network overhead in the IRS-aided systems, two signaling overheads, namely the pilot signaling overhead and control signaling overhead, are considered. Then, the practical achievable rate model considering two signaling overheads is newly defined as a performance metric of the discrete phase shift IRS systems. To maximize the achievable rate, the optimal resolution of discrete phase shift should be properly designed by finding the balance between the IRS passive beamforming gain and the amount of signaling overhead. Utilizing the concavity of the achievable rate model with respect to the discrete phase resolution, we propose the incremental search method to find the optimal resolution of the discrete phase shift. Once the IRS discrete phase shift resolution is chosen, the optimal discrete phase shift vector can be designed by a well-known branch-and-bound approach, whose computational complexity is exponential over the number of IRS phase shift elements and the discrete phase resolution. Therefore, two low-complexity alternative suboptimal methods are introduced, namely the greedy-based discrete phase shift and the quantized block coordinate descent (BCD) algorithms. From the simulation results, it is numerically shown that the optimal discrete phase shift resolution exists for a given transmit power and the system configurations, such as the number of IRS elements and the number of transmit antennas. Furthermore, it is verified that the proposed incremental search with discrete phase shift algorithms can improve the practical achievable rate considering the signaling overhead compared to the analog phase shift method by choosing the proper discrete phase shift resolution. Moreover, the performance comparisons between the greedy and the quantized BCD algorithms are provided under various system parameters. From this study, the merit and potential power of the discrete phase shift IRS technique are verified. Furthermore, the guidelines for choosing the optimal IRS phase shift resolution under various system parameters are provided. Specifically, the main contributions of this paper are summarized as follows:

- The practical achievable rate considering the pilot signal overhead and control signal overhead is modeled to evaluate the performance of the IRS systems adopting discrete phase shift methods.
- The concavity of the achievable rate over the IRS phase shift resolution is numerically shown. Following the results, the incremental search algorithm is proposed to obtain the optimal discrete phase shift resolution that can maximize the achievable rate.
- In the fixed discrete phase shift resolution, two suboptimal algorithms to find the optimal discrete phase shift values, namely the greedy and BCD algorithms, are introduced. From the simulation results, some meaningful observations on these two sub-algorithms are verified as follows:
  *Observation 1:* When *N* is relatively small, the BCD algorithm achieves a higher achievable rate than the greedy algorithm in a low signal-to-noise ratio (SNR) regime.
  *Observation 2:* When *N* is sufficiently large and $n_{t}$ is small, the greedy algorithm outperforms the BCD algorithms in a high SNR regime.
- This study verifies the merit of the IRS discrete phase shift method for spatial diversity systems. Providing the optimal resolution of IRS discrete phase shift under various system configurations, our work provides a guideline to design the phase shift resolution of IRS-aided multiple-antenna systems.

The remaining parts of this paper are organized as follows. The system and signal models of an IRS-aided system are introduced in Section 2. In Section 3, an overhead-aware achievable rate model is defined by using two signaling overhead parameters. In Section 4, a discrete phase shift resolution optimization problem is formulated, and an incremental-search-based algorithm is proposed to find the optimal discrete phase shift resolution. Here, two suboptimal methods are introduced to solve the subproblems with low complexity. Then, Section 5 is devoted to verifying the proposed algorithms by showing the numerically obtained optimal discrete phase shift resolution under various communication parameters and its practical achievable rate performances. Section 6 concludes this paper.

*Notations:* Superscripts *T*, *H*, ∗, and −1 denote the transposition, Hermitian transposition, complex conjugate, and inversion, respectively, for any scalar, vector, or matrix. The notations |x| and ∥x∥ denote the absolute value of *x* and the Euclidean norm of vector x, respectively; $I_{m}$ represents an m×m identity matrix; $0_{m\times n}$ and $1_{m\times n}$ denote the m×n zero and all-ones matrices, respectively; tr(X) is the trace operation of matrix X; diag(x) returns a diagonal matrix whose main diagonal elements are equal to x; $X_{m,n}$ represents the (m,n)th element of X; $x_{m}$ is the *m*th element of a vector x; |X| denotes the cardinality of set X; for a complex value *x*, Re{x} and Im{x} take the real and imaginary parts of *x*, respectively; ⊗ denotes the Kronecker product; and $x∼\mathrm{CN}(0,\sigma^{2})$ means that a complex random variable *x* conforms to a complex normal distribution with a mean of zero and variance $\sigma^{2}$. E[x] stands for the expectation value of random variable *x*.

## 2. System Model

**Figure 1:**
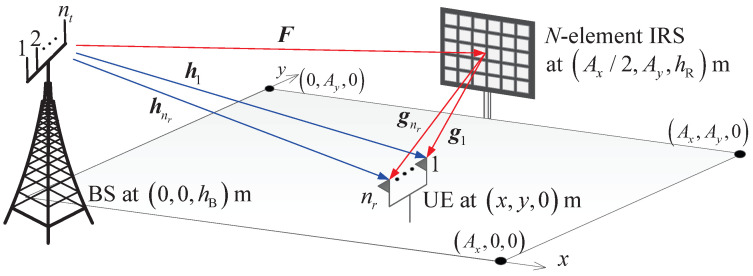
IRS-aided $n_{t}$-by-$n_{r}$ system model in $A_{x}$-by-$A_{y}$ area. A BS has $n_{t}$ antennas, an IRS has *N* elements ($N_{v}$-by-$N_{h}$), and a UE has $n_{r}$ antennas. The BS, IRS, and UE are denoted by B, R, and U, respectively.

An IRS-aided multiple-antenna system, in which a BS (or access point) transfers information to the user equipment (UE) with the aid of IRS with *N* elements, is considered as shown in Figure 1. The UE receives signals with $n_{r}$ antennas from one BS through an IRS, i.e., downlink transmission is considered. The rectangular coverage area is defined by $A_{x}\times A_{y}\;m^{2}$. The BS and IRS are located at $\left(0,0,h_{B}\right)$ and $\left(\frac{A_{x}}{2},A_{y},h_{R}\right)$, respectively, where $h_{B}$ and $h_{R}$ are the heights of the BS and IRS, respectively. The location of UE is denoted by (x,y,0). The transmitted signals are reflected by *N* reflection elements on an IRS and received by $n_{r}$ antennas at the UE. The IRS is linked to the BS via wireless channels, and the phase shift of each IRS element is controlled by the BS. To prevent clutter, we denote the BS, IRS, and UE by B, R, and U, respectively, in equations throughout the paper.

### 2.1. Channel Model

The direct channel from the *m*th antenna of BS to the *i*th receive antenna of the UE is modeled as a Rayleigh fading channel as

(1) $$h_{i,m}=\sqrt{\eta (d_{B,U})}{\bar{h}}_{i,m},$$

where ${\bar{h}}_{i,m}∼\mathrm{CN}\left(0,1\right)$ for $i\in N_{r}=\left\{1,\ldots ,n_{r}\right\}$ and $m\in N_{t}=\left\{1,\ldots ,n_{t}\right\}$. Here, $\eta (d_{a,b})$ denotes a path loss between a and b as a function of the distance between them, $d_{a,b}$ in which (a,b)∈{(B,R),(R,U)}. On the other hand, the IRS channels are modeled as a Rician channel, because the IRS is located at a distance of $h_{R}$ above the ground to ensure the line-of-sight (LoS), and usually the distance between the IRS and the UE is closer than that between the BS and the UE. The channel from the *m*th antenna of BS to the *n*th element of IRS is denoted by $f_{n,m}$ and its matrix representation is $F=\left[f_{1}\;\cdots \;f_{n_{t}}\right]\in C^{N\times n_{t}}$ and $f_{m}={\left[f_{1,m}\;\cdots \;f_{N,m}\right]}^{T}\in C^{N\times 1}$; the channel from the *n*th IRS element to the *i*th antenna at the UE is denoted by $g_{i,n}^{*}$ and its vector form is $g_{i}={\left[g_{i,1}\;\cdots \;g_{i,N}\right]}^{H}\in C^{N\times 1}$ in which $i\in N_{r}$. All the channels related to the IRS elements are modeled as the Rician channels as follows [57,58]:

(2a) $$\begin{array}{ccc} f_{m} & = & \sqrt{\eta (d_{B,R})}\left(\sqrt{\frac{K_{f}}{1+K_{f}}}f_{m}^{\mathrm{LoS}}+\sqrt{\frac{1}{1+K_{f}}}f_{m}^{\mathrm{NLoS}}\right)\in C^{N\times 1},\;m\in N_{t}, \end{array}$$

(2b) $$\begin{array}{ccc} g_{i} & = & \sqrt{\eta (d_{R,U})}\left(\sqrt{\frac{K_{g}}{1+K_{g}}}g_{i}^{\mathrm{LoS}}+\sqrt{\frac{1}{1+K_{g}}}g_{i}^{\mathrm{NLoS}}\right)\in C^{N\times 1},\;i\in N_{r}, \end{array}$$

where $K_{f}$ and $K_{g}$ are the Rician factors of $f_{m}$ and $g_{i}$, respectively; $f_{m}^{\mathrm{LoS}}$ and $g_{i}^{\mathrm{LoS}}$ denote the LoS components; while $f_{m}^{\mathrm{NLoS}}$ and $g_{i}^{\mathrm{NLoS}}$ denote the non-LoS (NLoS) components. The NLoS components are independent of one another and follow the standard complex Gaussian distribution with zero mean and unit variance, i.e., $f_{m}^{\mathrm{NLoS}}∼\mathrm{CN}\left(0,I_{N}\right)$ and $g_{i}^{\mathrm{NLoS}}∼\mathrm{CN}\left(0,I_{N}\right)$.

The IRS is assumed to be an $N_{v}$-by-$N_{h}$ rectangular uniform array in which $N_{v}$ and $N_{h}$ are the numbers of elements in each row and column of the array, respectively. The LoS channel between the *m*th antenna of BS and the IRS is modeled as follows [58]:

(3) $$f_{m}^{\mathrm{LoS}}=\exp\left(-\frac{j2\pi d}{\lambda_{W}}\left(m-1\right)\sin\theta_{\mathrm{AoD},B}\right)a_{v}^{*}\left(\theta_{\mathrm{AoA},R},\phi_{\mathrm{AoA},R}\right)⊗a_{h}^{*}\left(\theta_{\mathrm{AoA},R},\phi_{\mathrm{AoA},R}\right).$$

Here, $\theta_{\mathrm{AoA},R}$ and $\phi_{\mathrm{AoA},R}$ denote the center azimuth and elevation angle of arrival (AoA) at the IRS, and $a_{v}\left(\theta ,\phi \right)\in C^{N_{v}\times 1}$ and $a_{h}\left(\theta ,\phi \right)\in C^{N_{h}\times 1}$ are the phase steering vectors whose *n*th elements are defined as

(4a) $$\begin{array}{ccc} a_{v,n}\left(\theta ,\phi \right) & \;=\; & \exp\left(\frac{j2\pi d}{\lambda_{W}}\left(n-1\right)\cos\theta \cos\phi \right),\;n\in \left\{1,\ldots ,N_{v}\right\}, \end{array}$$

(4b) $$\begin{array}{ccc} a_{h,n}\left(\theta ,\phi \right) & \;=\; & \exp\left(-\frac{j2\pi d}{\lambda_{W}}\left(n-1\right)\sin\theta \cos\phi \right),\;n\in \left\{1,\ldots ,N_{h}\right\}, \end{array}$$

respectively, with the separation *d* between neighboring elements in the vertical and horizontal directions and the carrier wavelength $\lambda_{W}$.

Similarly, the LoS channel $g_{i}^{\mathrm{LoS}}\in C^{N\times 1}$ between the IRS and the *i*th antenna of the UE is modeled as follows [58]:

(5) $$g_{i}^{\mathrm{LoS}}=\exp\left(\frac{j2\pi d}{\lambda_{W}}\left(i-1\right)\sin\theta_{\mathrm{AoA},U}\right)a_{v}\left(\theta_{\mathrm{AoD},R},\phi_{\mathrm{AoD},R}\right)⊗a_{h}\left(\theta_{\mathrm{AoD},R},\phi_{\mathrm{AoD},R}\right),$$

where $\theta_{\mathrm{AoA},U}$ is the center azimuth AoA at the UE, and $\theta_{\mathrm{AoD},R}$ and $\phi_{\mathrm{AoD},R}$ denote the center azimuth and elevation angle of departure (AoD) from the IRS, respectively.

### 2.2. Signal Model

According to [44], an ideal IRS reflection coefficient can be modeled as a unit amplitude phase shifter, which can be expressed as $\psi_{n}=e^{j\chi_{n}}$, ∀n∈N={1,…,N}. Assuming that the analog phase shift values are available, i.e., $\chi_{n}\in \left[0,2\pi \right)$, ∀n∈N, the analog IRS phase shift vector is modeled as

(6) $$\begin{array}{ccc} \psi & = & {\left[\psi_{1}\;\psi_{2}\;\cdots \;\psi_{N}\right]}^{T}\\ & = & {\left[e^{j\chi_{1}}\;e^{j\chi_{2}}\;\cdots \;e^{j\chi_{N}}\right]}^{T}\in C^{N\times 1}. \end{array}$$

Practically, however, the IRS with analog phase shifters is difficult to apply due to the high IRS network overhead of delivering the IRS phase shift information from the BS to the IRS and the hardware implementation cost. In this study, the IRS with a discrete phase shift method, whose phase shift values can be represented in finite number of bits, is considered. Let the number of available phase shift values be denoted by $2^{b}$, then the *b*-bit resolution discrete IRS phase shift vector is modeled as

(7) $$\begin{array}{ccc} \psi_{b} & = & {\left[\psi_{b,1}\;\psi_{b,2}\;\cdots \;\psi_{b,N}\right]}^{T}\\ & = & {\left[e^{j\chi_{b,1}}\;e^{j\chi_{b,2}}\;\cdots \;e^{j\chi_{b,N}}\right]}^{T}\in C^{N\times 1}, \end{array}$$

where $\chi_{b,n}$ is chosen in the finite candidate phase set which is defined by

(8) $$F_{b}=\left\{0,\frac{1}{2^{b}}2\pi ,\frac{2}{2^{b}}2\pi ,\ldots ,\frac{2^{b}-1}{2^{b}}2\pi \right\}.$$

Here, every adjacent pair of discrete phase shifts has an equally spaced phase interval of $2^{1-b}\pi$. As the resolution of the IRS discrete phase shift *b* increases, the phase interval reaches zero, which can be interpreted as the analog phase shifts.

The effective overall composite channel vector, denoted by $\Omega_{i}={\left[\Omega_{i,1}\;\cdots \;\Omega_{i,n_{t}}\right]}^{H}\in C^{n_{t}\times 1}$ at the *i*th antenna of the UE, is written as follows:

(9) $$\begin{array}{ccc} \Omega_{i}^{H} & \;=\; & h_{i}^{H}+\left[\begin{array}{ccc} \underset{n\in N}{\sum }g_{i,n}^{*}e^{j\chi_{b,n}}f_{n,1} & \cdots & \sum_{n\in N}g_{i,n}^{*}e^{j\chi_{b,n}}f_{n,n_{t}} \end{array}\right]\\ & = & h_{i}^{H}+g_{i}^{H}\mathrm{diag}\left(\psi_{b}\right)F,\;i\in N_{r}, \end{array}$$

where $h_{i}={\left[h_{i,1}\;\cdots \;h_{i,n_{t}}\right]}^{H}\in C^{n_{t}\times 1}$.

After the CSI acquisition, which will be presented in the next section, the full CSI, such as $h_{i}$, F, and $g_{i}$, can be obtained at the BS. The IRS phase shift vector is then calculated by the BS. Hence, the composite channel vector $\Omega_{i}$ can be easily obtained from (9). Therefore, the spatial diversity schemes utilizing full CSI only at the transmitter, namely maximum ratio transmission (MRT) and space–time line code (STLC), can be applied to the IRS-aided multiple-antenna systems (To avoid additional CSI acquisition procedures, the spatial diversity schemes utilizing the full CSI at the UE, e.g., the maximum ratio combining [59] and space–time block code (STBC) [60], are not considered in this paper). Here, STLC is a dual scheme to the STBC that encodes the information symbols with full CSI at the transmitter and decodes them by simply combining the received signals with an extremely limited CSI at the receiver [61,62] (Owing to the beneficial and unique features of the STLC schemes, such as low computational complexity, full-diversity gain, and high scalability for the number of transmit antennas, the STLC technique has been vigorously applied to various multi-antenna communication systems. For example, multiuser/multicast/multi-stream multiplexing systems [62,63], cooperative communication systems [64], OFDM systems [65], mobile communication systems [66,67], secure wireless communication systems [68], and over-the-air computation systems [69]. Moreover, the statistical properties of SNR and signal-to-interference-plus-noise ratio and the performance of STLC, namely bit-error-rate and achievable rate, were analyzed in [70,71]). According to [61], the received SNR of the MRT and STLC can be generalized as follows:

(10) $$\begin{array}{c} \mathrm{SNR}=\frac{\gamma_{n_{t}n_{r}}P}{n_{r}C\sigma_{z}^{2}}, \end{array}$$

where *P* and $\sigma_{z}^{2}$ are the power of the transmit signal and additive white Gaussian noise, respectively; *C* is a code rate depending on the transmission scheme and antenna configurations [61]. The real-valued effective channel gain is written as follows:

(11) $$\begin{array}{c} \gamma_{n_{t}n_{r}}=\sum_{m=1}^{n_{t}}\sum_{i=1}^{n_{r}}{\left|\Omega_{i,m}\right|}^{2}. \end{array}$$

From (10), it can be seen that the received SNR maximization of the IRS-aided multiple-antenna system is equivalent to maximizing the effective channel gain when the transmission scheme is chosen at the BS. Therefore, we concentrate on maximizing the effective channel gain in IRS phase shift design in the latter part of the paper.

## 3. Achievable Rate Model for IRS-Aided Multiple-Antenna Systems

To benefit from the IRS-aided multiple-antenna system, the CSI should be known at the BS to design the precoder and the IRS phase shift vector. Then, the IRS phase shift values designed from BS are transmitted to the IRS to control the phase value of each element. Here, the network overhead occurring from the channel estimation and IRS phase value transmission can deteriorate the communication performance. In this section, the achievable rate model is newly defined by introducing the CSI acquisition scenario and the two signaling overheads of the IRS system.

### 3.1. CSI Acquisition Scenario

As reported in [72,73,74], however, accurate and low-overhead channel estimation in IRS-based systems is one of the most critical challenges. Specifically, $\Omega_{i}$ in (9) is required at the BS for the proposed IRS-aided system. Here, the effective channel $\Omega_{i}$ can be obtained from the knowledge of (i) direct-link channels $h_{i}$ between BS and the *i*th antenna at UE; (ii) indirect-link channels $g_{i,n}^{*}f_{n,m},\forall n\in N$ and $i\in N_{r}$; and (iii) the phase shift values $\chi_{n}$ (or $\chi_{b,n}$), ∀n∈N. Since the phase shift values are designed from $h_{i}$ and $g_{i,n}^{*}f_{n,m}$, which will be shown in Section 4, we introduce an example framework to estimate $h_{i}$ and $g_{i,n}^{*}f_{n,m}$.

As the first step, the direct-link channel $h_{i}$ is estimated by sending an orthogonal sounding pilot from the *i*th antenna of UE to the BS while all IRS elements are turned off. In the second step, the BS estimates the indirect-link channels from the cascaded channels $g_{i,n}^{*}f_{n,m}$ by using orthogonal pilots [29,75,76]. Specifically, since the cascaded channel $g_{i,n}^{*}f_{n,m}$ is linked with the *n*th IRS element, all IRS elements except the *n*th IRS element are turned off, and the phase shift value of the *n*th IRS element is set to zero. For a given $i\in N_{r}$, repeating the cascaded channel estimation for all *n*, $\mathrm{diag}{\left(g_{i}\right)}^{H}f_{m}$ can be obtained. Therefore, a UE sends at least $n_{r}\left(N+1\right)$ sounding pilot symbols in training duration because the BS estimates $n_{r}\left(N+1\right)$ parameters, namely $h_{i}$ and $g_{i,n}^{*}f_{n,m}$ for all n∈N and $i\in N_{r}$.

In static channels, the cascaded channel estimation approach is preferred to an individual channel estimation approach that separately estimates the channels $g_{i}$ and $f_{m}$ [77,78], because the number of parameters to be estimated is smaller in the cascaded channel than that in two individual channels. Under time-varying channels, however, the number of pilots (i.e., the length of training duration) can be reduced by employing the individual channel estimation approach. In this case, a UE is a mobile terminal whereas a BS and IRS are typically fixed, and the coherence time of a BS-IRS link for $f_{m}$ is much longer than that of an IRS-UE link for $g_{i}$. Thus, the BS estimates $f_{m}$ less frequently than $g_{i}$, and accordingly, the pilot overhead can be reduced. In this study, the channels are assumed to be static, and therefore, the cascaded channel estimation approach is employed. The whole training (i.e., estimation and signaling) procedure to obtain $h_{i}$ and $g_{i,n}^{*}f_{n,m}$ is summarized as follows:

Step 1: The user sends pilot symbols (training sequence) using the *i*th antenna while all IRS elements are turned off.
Step 2: The BS estimates the direct channel $h_{i}$.
Step 3: The user sends pilot symbols (training sequence) using the *i*th antenna while the *n*th IRS element is turned on with $\chi_{n}=0$ and other IRS elements are turned off.
Step 4: The BS estimates the indirect cascaded channel $g_{i,n}^{*}f_{n,m}$.
Step 5: Repeat Steps 3 and 4 from n=1 to n=N.
Step 6: Repeat Steps 1–5 from i=1 to $i=n_{r}$.

More sophisticated channel estimation algorithms can be developed. However, the main focus of this paper is not on channel estimation but on IRS phase shift design. In this study, therefore, a simple channel sounding and estimation algorithm is considered.

### 3.2. Network Overheads and Achievable Rate Models for Discrete Phase Shift IRS Systems

Assume that there is no dedicated control channel for sharing the phase shift values in the BS-IRS link. We then model the achievable rate considering the network signaling overhead for IRS phase shifts. Denoting the downlink communication duration and the operation bandwidth as *T* and *W*, respectively, the downlink achievable rate can be evaluated as follows:

(12) $$R=W\left(\frac{T-t_{p}-t_{c}}{T}\right)\log_{2}\left(1+\frac{\gamma_{n_{t}n_{r}}P}{n_{r}C\sigma_{z}^{2}}\right)\;\left[\mathrm{bits}/\sec\right],$$

where $t_{p}$ is the training duration for the pilot signaling; and $t_{c}$ is the controlling time of IRS, i.e., the time overhead for transferring the phase shift values from the BS to the IRS which depends on the IRS phase shift design schemes. Since the channel estimation for a communication link with IRS requires at least $n_{r}\left(N+1\right)$ pilot transmissions from the user, as discussed in Section 3.1, $t_{p}$ with IRS is greater than $t_{p}$ without IRS which requires only $n_{r}$ pilot transmissions to estimate $h_{i}$ where $i\in N_{r}$. Considering the pilot transmission time, we model the training duration for channel estimation as below:

(13) $$t_{p}=\xi n_{r}\left(N+1\right)t_{s}\;\left[\sec\right],$$

where $t_{s}$ is a sampling duration that can be modeled as reciprocal of the bandwidth, i.e., $t_{s}=1/W$; ξ is the scaling factor depending on the pilot overhead (ξ≥1). To improve the CSI estimation accuracy, we can increase ξ greater than one. On the other hand, as IRS phase shift value $\chi_{b,n}$ can be expressed as *b* bits, and the total bN controlling bits should be transferred for the IRS phase shifts. Assuming that (i) $b_{Q}$ bits are modulated by a symbol, i.e., the modulation size of $2^{b_{Q}}$; and (ii) there is no error for the IRS phase shift information transfer (i.e., the BS-IRS link is error-free for the IRS control signals), and $t_{c}$ can be defined as

(14) $$t_{c}=\frac{bN}{b_{Q}}t_{s}\;\left[\sec\right].$$

By substituting (13) and (14) into (12), the practical downlink achievable rate of the IRS systems can be written as

(15) $$R\left(\psi_{b},b\right)=W\left(1-\frac{\xi n_{r}\left(N+1\right)t_{s}+\frac{bN}{b_{Q}}t_{s}}{T}\right)\log_{2}\left(1+\frac{\gamma_{n_{t}n_{r}}P}{n_{r}C\sigma_{z}^{2}}\right)\;\left[\mathrm{bits}/\sec\right].$$

Then, the overhead-aware achievable rate maximization problem can be formulated as

(16a) $$\begin{array}{ccc} & \max_{\{\chi_{1},\ldots ,\chi_{N}\},b}\; & R\left(\psi_{b},b\right) \end{array}$$

(16b) $$\begin{array}{ccc} & s.t. & \psi_{b}={\left[e^{j\chi_{1}}\cdots e^{j\chi_{N}}\right]}^{T}\in C^{N\times 1}, \end{array}$$

(16c) $$\begin{array}{ccc} & & \chi_{n}\in F_{b},\forall n\in N,b\in \left\{1,\ldots ,b_{\max}\right\}. \end{array}$$

Here, $b_{\max}$ denotes the maximum value of the discrete phase shift whose phase shift resolution is sufficiently high so that the phase interval $2^{1-b_{\max}}\pi ≃0$. In this study, the $b_{\max}$-bit discrete phase shift is assumed to achieve identical performance to the analog phase shift in (6) with negligible power loss.

## 4. IRS Discrete Phase Shift Vector Design with Optimal Phase Shift Resolution

Unfortunately, the problem (16) is a high-dimensional combinatorial problem whose search complexity is given as $\sum_{b=1}^{b_{\max}}{\left|F_{b}\right|}^{N}=\sum_{b=1}^{b_{\max}}2^{bN}$. Specifically, when the discrete phase resolution *b* is given, the set of discrete phase value $F_{b}$ is determined so that the *b*-bit optimal phase shift vector $\psi_{b}$ can be obtained among a total of $2^{bN}$ combinations. To find the optimal phase shift resolution, the concavity of (16a) over *b* is first numerically presented in this section. Then, utilizing the concavity of the achievable rate model, the incremental search method is proposed by fixing the resolution of the discrete phase shift from b=1 to the higher resolution.

When *b* is fixed to constant, the objective function (16a) is only a function of the IRS phase shift vector $\psi_{b}$. As the logarithmic function is a monotonically increasing function, (16) is reduced to a problem of maximizing the real-valued effective channel gain $\gamma_{n_{t}n_{r}}$ with a *b*-bit discrete phase shift, which is formulated as follows:

(17a) $$\begin{array}{ccc} & \max_{\left\{\chi_{1},\ldots ,\chi_{N}\right\}} & \gamma_{n_{t}n_{r}} \end{array}$$

(17b) $$\begin{array}{ccc} & s.t. & \psi_{b}={\left[e^{j\chi_{1}}\cdots e^{j\chi_{N}}\right]}^{T}\in C^{N\times 1} \end{array}$$

(17c) $$\begin{array}{ccc} & & \chi_{n}\in F_{b},\forall n\in N,b\in Z^{+}. \end{array}$$

Here, the effective channel gain $\gamma_{n_{t}n_{r}}$ can be expressed as a product of an IRS reflecting vector and an effective channel matrix as follows:

(18) $$\begin{array}{ccc} \gamma_{n_{t}n_{r}} & = & \sum_{i=1}^{n_{r}}{∥\Omega_{i}∥}^{2}\\ & = & \sum_{i=1}^{n_{r}}{∥h_{i}+F^{H}\mathrm{diag}{\left(\psi_{b}\right)}^{H}g_{i}∥}^{2}\\ & = & \sum_{i=1}^{n_{r}}{∥h_{i}+F^{H}\mathrm{diag}\left(g_{i}\right)\psi_{b}^{*}∥}^{2}\\ & = & \sum_{i=1}^{n_{r}}\left[\begin{array}{cc} \psi_{b}^{T} & 1 \end{array}\right]\left[\begin{array}{c} \mathrm{diag}{\left(g_{i}\right)}^{H}F\\ h_{i}^{H} \end{array}\right]{\left[\begin{array}{c} \mathrm{diag}{\left(g_{i}\right)}^{H}F\\ h_{i}^{H} \end{array}\right]}^{H}\left[\begin{array}{c} \psi_{b}^{*}\\ 1 \end{array}\right]\\ & = & \left[\begin{array}{cc} \psi_{b}^{H} & 1 \end{array}\right]M_{c}{\left[\begin{array}{cc} \psi_{b}^{H} & 1 \end{array}\right]}^{H}. \end{array}$$

Here, $M_{c}\in C^{\left(N+1)\times (N+1\right)}$ is the complex-valued effective channel matrix of the IRS-aided $n_{t}\times n_{r}$ system defined as

(19) $$M_{c}≜{\left[\begin{array}{cc} \sum_{i\in N_{r}}\;\;\;\;\mathrm{diag}{\left(g_{i}\right)}^{H}FF^{H}\mathrm{diag}\left(g_{i}\right) & \sum_{i\in N_{r}}\;\;\;\;\mathrm{diag}{\left(g_{i}\right)}^{H}Fh_{i}\\ \sum_{i\in N_{r}}\;h_{i}^{H}F^{H}\mathrm{diag}\left(g_{i}\right) & \sum_{i\in N_{r}}\;h_{i}^{H}h_{i} \end{array}\right]}^{*}.$$

However, (17) is still a non-linear integer program, whose optimal solution can be obtained by applying the branch-and-bound algorithm [44]. Due to its NP-hardness, however, the worst-case complexity of the branch-and-bound method exponentially increases over *N* and *b*. Therefore, to reduce the computational complexity, the two general IRS discrete phase shift design approaches are introduced as follows.

One simplest approach to obtain the suboptimal solution of (17) is directly quantizing the optimal analog phase to the nearest discrete phase value among the finite discrete phase set $F_{b}$. Once the analog phase shift vector $\tilde{\psi }={\left[e^{{\tilde{\chi }}_{1}}\;e^{{\tilde{\chi }}_{2}}\cdots e^{{\tilde{\chi }}_{N+1}}\right]}^{T}\in C^{(N+1)\times 1}$ is obtained from various existing methods, the *b*-bit uniform quantization is applied by mapping the nearest phase value among $F_{b}$ as follows:

(20) $$\chi_{n}^{☆}=\underset{\chi \in F_{b}}{\arg\min}\left|\chi -{\tilde{\chi }}_{n}\right|,\;\forall n\in N.$$

From (20), the *b*-bit discrete phase shift vector $\psi_{b}^{☆}$ is obtained. Here, various existing algorithms can be adopted to obtain the IRS analog phase shift, such as semidefinite relaxation [5], an alternating direction method of multiplier [79], manifold optimization [47], unit-modulus constraint relaxation (UCR) [80], and BCD [81]. In this study, the analog phase shift values obtained from the BCD algorithm are uniformly quantized by (20), the so-called quantized BCD algorithm in this study. The overall quantized BCD method is summarized in Algorithm 1. Here, $I_{c}$ is a maximum iteration number to guarantee the convergence of the BCD algorithm, which in this study, is set to $I_{c}=5$. The more detailed information about the convergence of the BCD algorithm can be verified in [81].

Introducing another approach called greedy-based method, the phase shift values from the first to the *N*th IRS elements are sequentially determined through *N* greedy steps. While the best phase shift values determined in the previous greedy steps are fixed, the best phase shift value in the current greedy step is selected from $F_{b}$ in (8) such that the objective function is maximized. The greedy-based algorithm is summarized in Algorithm 2.


**Algorithm 1** Quantized BCD-Based IRS Discrete Phase Shift Algorithm.
1:**Input**: Effective channel matrix $M_{c}$ and the discrete phase shift resolution *b*.2:**Output**: *b*-bit resolution discrete phase shift vector $\psi_{b}\in C^{N\times 1}$.3:Initialization: ${\hat{\psi }}^{\left(0\right)}=1_{(N+1)\times 1}$.4:
**for** i=1 **to** $I_{c}$ **do**
5:    ${\bar{\psi }}^{\left(i\right)}\;=\;M_{c}{\hat{\psi }}^{\left(i-1\right)}\in C^{(N+1)\times 1}$.6:    ${\hat{\psi }}_{n}^{\left(i\right)}=\frac{{\bar{\psi }}_{n}^{\left(i\right)}}{|{\bar{\psi }}_{n}^{\left(i\right)}|},\forall n\in \left\{1,\ldots ,N+1\right\}$.7:
**end for**
8:Analog phase shift vector $\tilde{\psi }={\hat{\psi }}^{\left(I_{c}\right)}/{\hat{\psi }}_{N+1}^{\left(I_{c}\right)}$.9:Set $F_{b}$ in (8)10:
**for** n=1 **to** *N* **do**
11:    $\chi_{n}^{☆}=\underset{\chi \in F_{b}}{\arg\min}\left|\chi -{\tilde{\chi }}_{n}\right|,\;\forall n\in N$12:
**end for**
13:**Return**$\psi_{b}={\left[e^{j\chi_{1}^{☆}}\;\;e^{j\chi_{2}^{☆}}\;\;\cdots \;\;e^{j\chi_{N}^{☆}}\right]}^{T}\in C^{N\times 1}$.




**Algorithm 2** Greedy-Based IRS Discrete Phase Shift Algorithm.
1:**Input**: Effective channel matrix $M_{c}$ and the discrete phase shift resolution *b*.2:**Output**: *b*-bit resolution discrete phase shift vector $\psi_{b}\in C^{N\times 1}$.3:Initialization: $\chi_{n}=0,\forall n\in N$.4:Set $F_{b}$ in (8).5:
**for** n=1 **to** *N* **do**
6:    $\chi_{n}^{☆}=\underset{\chi_{n}\in F_{b}}{\arg\min}\left[e^{j\chi_{1}}\;\cdots \;e^{j\chi_{n}}\;\cdots \;e^{j\chi_{N}}\;1\right]M_{c}{\left[e^{j\chi_{1}}\;\cdots \;e^{j\chi_{n}}\;\cdots \;e^{j\chi_{N}}\;1\right]}^{H}$7:$\chi_{n}\leftarrow \chi_{n}^{☆}$.8:
**end for**
9:**Return**$\psi_{b}={\left[e^{j\chi_{1}^{☆}}\;\;e^{j\chi_{2}^{☆}}\;\;\cdots \;\;e^{j\chi_{N}^{☆}}\right]}^{T}\in C^{N\times 1}$.



**Figure 2:**
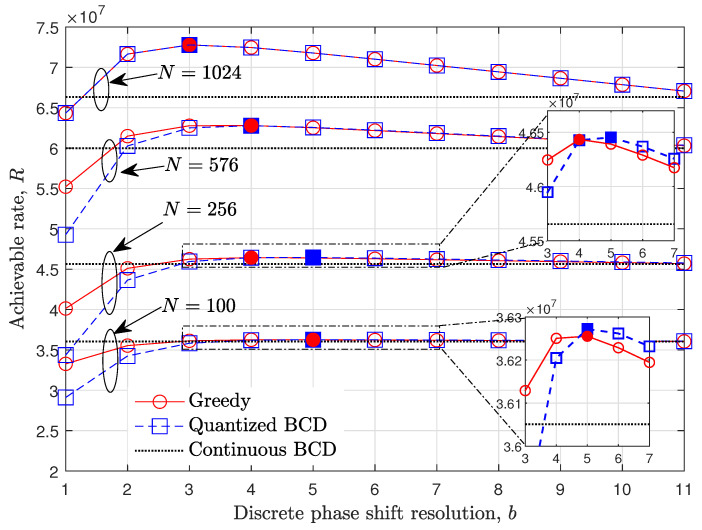
Achievable rate evaluation over the discrete phase shift resolution, *b*, for N∈{100,256,576,1024} when UE is at (400,200,0)m, P=30dBm and STLC transmission with $n_{t}=1$, $n_{r}=2$ is adopted.

**Figure 3:**
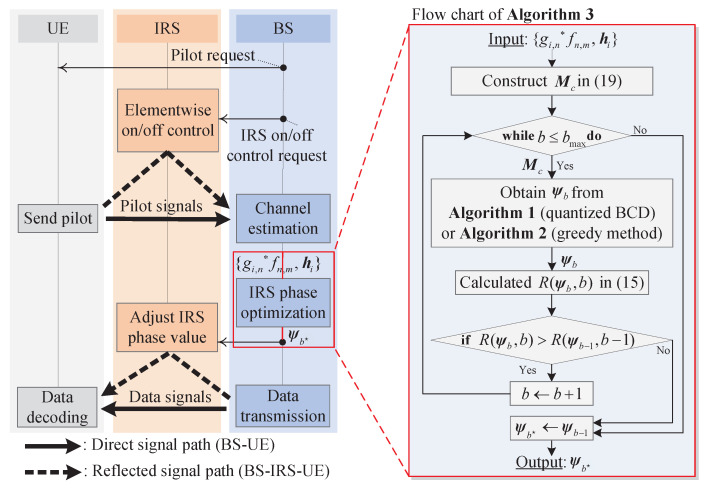
Overall system procedures and the flow chart of the IRS-aided systems.

Since the achievable rate for the fixed discrete phase shift resolution can be calculated using $\psi_{b}$ obtained from Algorithms 1 or 2, the optimal *b* can be found from the various line search algorithms. From (15), it can be easily seen that the trade-off between the IRS passive beamforming gain and signaling overhead exists. Specifically, as *b* increases, the effective channel gain $\gamma_{n_{t}n_{r}}$ increases by precisely expressing the optimal phase shift value of each IRS element. As opposed to the $\gamma_{n_{t}n_{r}}$, the data transmission time, i.e., $T-t_{p}-t_{c}$ in (15), linearly decreases as *b* increases due to the increase in IRS control signal overhead $t_{c}$. In other words, the (15) is expected to be a concave function with respect to *b*. Figure 2 is provided to show the concavity of an achievable rate *R* over *b* for N∈{100,256,576,1024} when the STLC transmission with $n_{t}=1$, $n_{r}=2$, and P=30dBm is considered (Here, the concavity of the discrete phase shift resolution *b* on achievable rate *R* can be analytically derived when IRS is deployed in the single-input single-output systems without the direct path between the BS and UE as [44,45]. However, when the direct path is considered, the derivations are not directly extended to multiple-antenna systems. Instead, the impact of discrete phase shift resolution is provided numerically for the various parameters). Here, the achievable rate of the analog phase shift method is calculated in fixed $b=b_{\max}≜12$. The marker filled inside shows the optimal discrete phase shift resolution $b^{☆}$ for each method. For example, when N=100, the optimal resolution $b^{☆}$ is 5, and $b^{☆}$ monotonically decreases as *N* increases. From the results, it is expected that the $b^{☆}$ can be obtained by searching $b^{☆}$ in the incremental search direction. Specifically, by increasing the integer-valued *b* from one, the achievable rate is calculated unless the terminate condition $R\left(\psi_{b},b\right)\leq R\left(\psi_{b-1},b-1\right)$ is satisfied. When the condition is satisfied, the $b^{☆}$ is chosen as the previous resolution value, i.e., b−1, which is the optimal point if the concavity of (15) holds. The incremental search method to obtain an optimal IRS discrete phase shift resolution is summarized in Algorithm 3. The overall system procedures of the IRS systems and flow chart of the proposed incremental search-based method are given in Figure 3.

## 5. Performance Evaluation and Discussion

In this section, the optimal discrete phase shift resolution $b^{☆}$ obtained by the proposed incremental search method is presented under various communication parameters. Furthermore, the achievable rate performance of the optimal discrete phase shift IRS is evaluated and compared to the analog phase shift method. During the simulations, the STLC encoding is applied as a transmit diversity scheme at the BS. The number of receiving antennas is set to two unless stated otherwise, so that the full rate transmission is available, i.e., C=1 [61]. Furthermore, $b_{\max}$ is set to 12, and the analog phase shift values are assumed to be quantized in 12-bit resolution to be transferred to IRS. The simulations are conducted under the parameters used in [81], which are described in Table 1 (The azimuth and elevation angles in Table 1 can be obtained using the location of BS, IRS, and UE, which are (0,0,10),(250,250,5), and (400,200,0),m, respectively. The detailed procedures are omitted in this paper, [82] is left for interested readers). For the variety of performance comparisons, the quantized UCR-based discrete phase shift algorithm [80] is additionally provided during the simulations.

**Figure 4:**
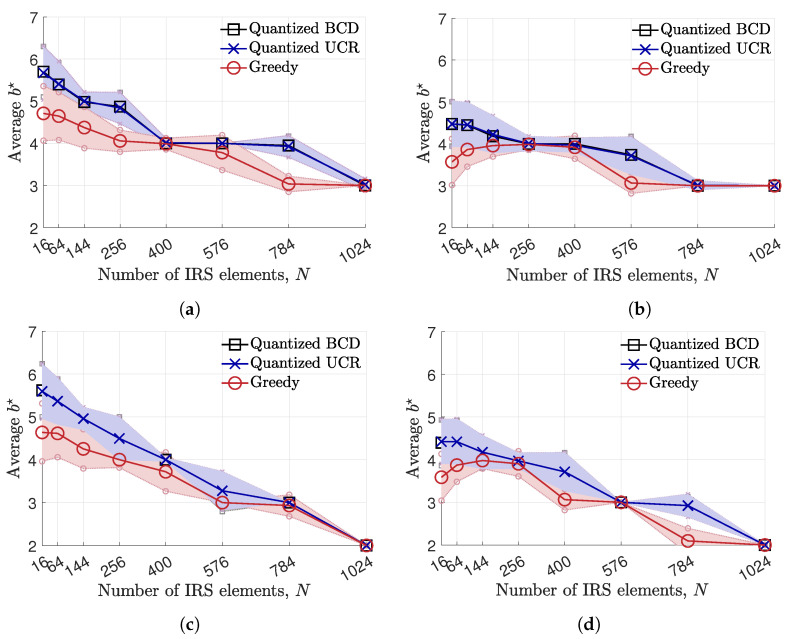
Average $b^{☆}$ across the number of IRS elements, *N*, when UE is at (400,200,0)m, $n_{r}=2$ and P=25dBm. (**a**) $n_{t}=1$ and ξ=1. (**b**) $n_{t}=8$ and ξ=1. (**c**) $n_{t}=1$ and ξ=40. (**d**) $n_{t}=8$ and ξ=40.

Following Algorithm 3, the average optimal discrete phase shift resolution $b^{☆}$ for each number of IRS element *N* is demonstrated in Figure 4, when $n_{r}=2,\;n_{t}\in \left\{1,8\right\},\;\xi \in \left\{1,40\right\}$ and P=25dBm. The error bar shows the average value of $b^{☆}$ (solid line) and the interval of unit-standard deviation (the region between upper and lower dotted lines) for each phase shift method. From the results, the average $b^{☆}$ obtained from the quantized BCD and UCR is generally higher than that obtained from the greedy algorithm. When *N* is large, e.g., N=1024, the $b^{☆}$ values obtained from every suggested method converge to three when ξ=1. However, when the pilot overhead is severe, i.e., ξ=40, the $b^{☆}$ is reduced to two, so as to alleviate the effect of pilot overhead. When $n_{t}=1$, the average $b^{☆}$ decreases as the number of IRS element *N* increases, as shown in Figure 2. This is because the smaller *b* is chosen to mitigate the IRS control signal overhead $t_{c}$ in (14). However, when $n_{t}=8$, the average $b^{☆}$ of the greedy algorithm adopts a lower $b^{☆}$ when N≤144. On the other hand, it is observed that the quantized BCD and UCR algorithms obtain almost the same average value of $b^{☆}$, while the BCD shows a slightly lower variance in choosing $b^{☆}$ compared to the UCR algorithm.
**Algorithm 3** Incremental Search-Based Discrete Phase Shift Resolution Finding Algorithm.1:**Input**: Channel state information $g_{i,n}^{*}f_{n,m},h_{i},\;\forall i,n,m$, the maximum discrete phase shift resolution $b_{\max}$, and communications parameters *T*, *W*, *P*, $t_{p}$, $t_{c}$.2:**Output**: The optimal discrete phase shift resolution $b^{☆}$ and corresponding phase shift vector $\psi_{b^{☆}}\in C^{N\times 1}$.3:Initialization: b=1.4:Compute $M_{c}$ in (19).5:**while**$b\leq b_{\max}$**do**6:    Set $F_{b}$ in (8).7:    Obtain $\psi_{b}$ from **Algorithms** 1 or 2.8:    Calculate $R\left(\psi_{b},b\right)$.9:    **if** $R\left(\psi_{b},b\right)>R\left(\psi_{b-1},b-1\right)$ **or** b=1 **then**10:        b←b+1.11:    **else**12:        **end while**13:    **end if**14:**end while**15:**Return**$b^{☆}=b-1$ and $\psi_{b^{☆}}=\psi_{b-1}\in C^{N\times 1}$.

**Figure 5:**
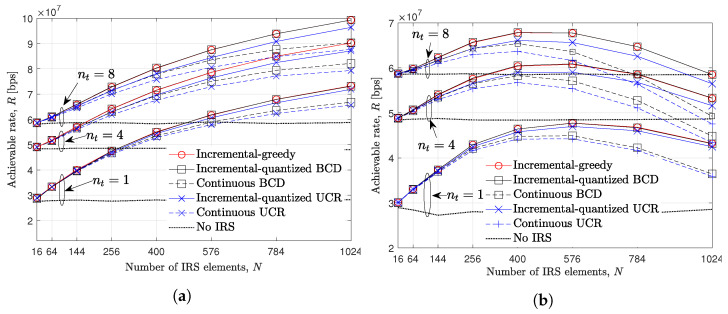
Achievable rate evaluation over the number of IRS elements, *N*, when UE is at (400,200,0)m, $n_{t}\in \left\{1,4,8\right\}$, $n_{r}=2$, P=25dBm. (**a**) ξ=1. (**b**) ξ=20.

In Figure 5, the achievable rate *R* over the number of IRS element *N* is evaluated when $n_{t}\in \left\{1,4,8\right\},n_{r}=2$, P=25dBm, and ξ∈{1,20}. The huge performance gap between no IRS and other methods verifies the advantages of deploying IRS. Furthermore, regardless of $n_{t}$, it is verified that the performance of the greedy and quantized BCD-based discrete phase shift algorithm achieve the highest achievable rate compared to the quantized UCR algorithm and the existing analog phase shift method. Here, note that the discrete phase shifts always show better performance than the analog phase shift method due to the amount of network overhead (13) and (14). In other words, discrete phase shift methods can significantly reduce the amount of overhead with affordable degradation on the IRS passive beamforming gain compared to the analog phase shift method. For example, when N=1024 and $n_{t}=8$, about 9Mbps of the performance gap between the incremental-quantized BCD and the existing analog BCD methods. When the pilot overhead is relatively small, i.e., ξ=1, the achievable rate from every method increases as *N* increases. Although the optimal discrete phase shift is considered, the achievable rate is degraded as *N* increases when ξ=20. For example, when $n_{t}=4$, the optimal number of IRS elements is 576 for the discrete phase shift methods, while it is 400 for the analog phase shift methods. On the other hand, the performance of the UCR method is severely degraded as *N* and $n_{t}$ increase due to its suboptimality which stems from fulfilling the relaxed constraint, as stated in [80].

**Figure 6:**
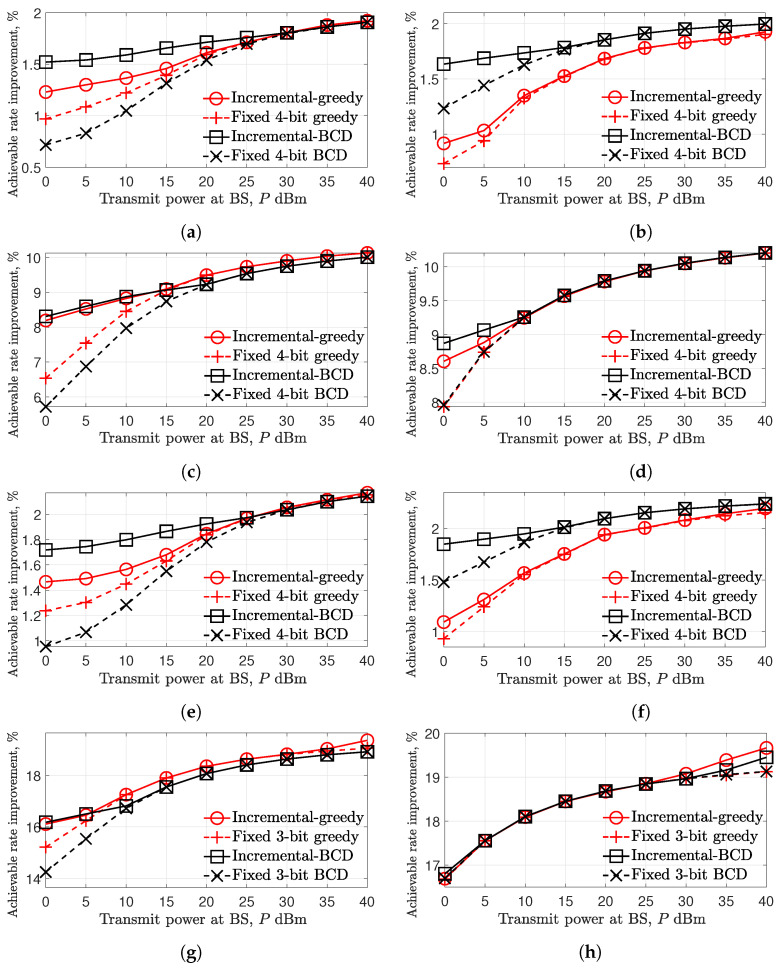
Achievable rate improvement over the transmit power at BS, *P*, when UE is at (400,200,0)m. (**a**) With 256-element IRS, $n_{t}=1$, and ξ=1. (**b**) With 256-element IRS, $n_{t}=8$, and ξ=1. (**c**) With 1024-element IRS, $n_{t}=1$, and ξ=1. (**d**) With 1024-element IRS, $n_{t}=8$, and ξ=1. (**e**) With 256-element IRS, $n_{t}=1$, and ξ=20. (**f**) With 256-element IRS, $n_{t}=8$, and ξ=20. (**g**) With 1024-element IRS, $n_{t}=1$, and ξ=20. (**h**) With 1024-element IRS, $n_{t}=8$, and ξ=20.

In Figure 6, the achievable rate improvement with a baseline of BCD-based analog phase shift method is evaluated over *P* when $n_{r}=2$, N∈{256,1024}, $n_{t}\in \left\{1,8\right\}$, and ξ∈{1,20}. Here, the performance of the UCR algorithm is omitted due to its degraded performance compared to the BCD algorithm verified in Figure 5. Instead, the discrete phase shift with fixed 3 and 4-bit methods are compared with the proposed incremental-based method. From the results, it is verified that the discrete phase shift methods are expected to obtain large achievable rate improvement as *N* and ξ increases. For example, in Figure 6h, more than 16% of the achievable rate is improved compared to the analog phase shift method. Interestingly, the superiority between two-sub algorithms, i.e., greedy and BCD algorithms, can be pointed out as follows:

**Remark** **1.** *When N is relatively small, e.g., N=256, the BCD algorithm outperforms the greedy algorithm, especially in a low-SNR regime (from Figure 6a,b,e,f).*

This is especially the case in Figure 6b, when $n_{t}=8$, and the quantized BCD algorithm outperforms the greedy algorithm in the entire SNR range.

**Remark** **2.** *When N is relatively large and $n_{t}$ is small, e.g., N=1024 and $n_{t}=1$, the greedy algorithm shows better performance compared to the quantized BCD algorithms in high SNR regime (from Figure 6c,g).*

For the case when N=1024 and $n_{t}=8$, which corresponded to Figure 6d,h, two sub-algorithms present comparable performance in the SNR range between 10 and 25dBm while showing their superiority in the low- and high-SNR regimes in Figure 6d,h, respectively. From the remarks, the sub-algorithms can be effectively chosen according to the received signal power level and the numbers of IRS elements, *N*, and transmit antennas, $n_{t}$.

**Figure 7:**
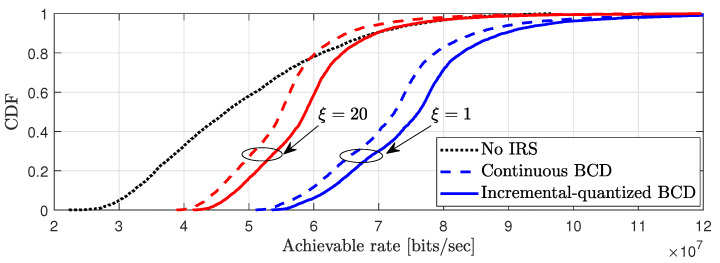
CDF of achievable rate performance for randomly generated 4000 UEs in $300\times 200\;m^{2}$ area. N=576, P=25dBm and ξ∈{1,20}.

To demonstrate the performance improvement of the IRS discrete phase shift method for the entire coverage area, the 4000 UEs are randomly generated to uniformly cover the two-dimensional coverage area in Figure 7. Specifically, the UEs are generated in the $300\times 200\;m^{2}$ area in the x axis range of x∈[100,400]m and y axis range of y∈[50,250]m to effectively show the performance of IRS. It is seen that the achievable rate is small when ξ=20, compared to when ξ=1. Specifically, at CDF=0.6, approximately 0.5bits/s improvements are accomplished in quantized BCD compared to the analog BCD method for both ξ=1 and ξ=20. Interestingly, when the pilot overhead is severe, i.e., ξ=20, it is shown that the performance of the IRS with analog BCD does not always outperform the systems without the IRS. This is because the effect of the IRS network overhead is larger than the IRS beamforming gain when the analog phase shift method is used. However, by properly choosing the discrete phase resolution, the quantized BCD method can provide considerable performance enhancement in the multiple antenna systems.

**Figure 8:**
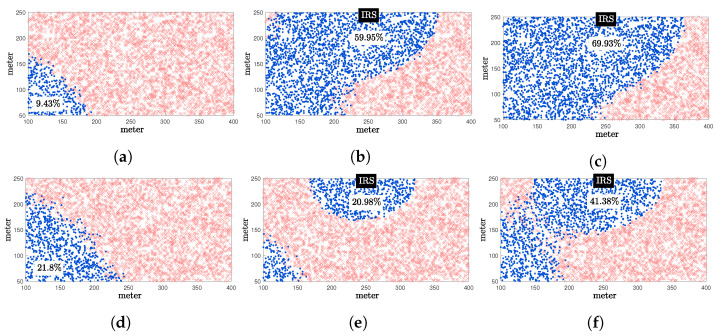
Spatial distributions of randomly generated 4000 UEs when $n_{t}=1$, $n_{r}=2$, and P=25dBm. Dot marks ‘·’ for UEs satisfying the target rate; Cross marks ‘×’ for UEs achieving achievable rate less than the target rate. (**a**) Without IRS, target rate of 7bits/sec/Hz and ξ=1. (**b**) N=576-element IRS with BCD method, target rate of 7bits/s/Hz and ξ=1. (**c**) N=576-element IRS with quantized BCD method, target rate of 7bits/sec/Hz and ξ=1. (**d**) Without IRS, target rate of 6bits/s/Hz and ξ=20. (**e**) N=576-element IRS with BCD method, target rate of 6bits/s/Hz and ξ=20. (**f**) N=576-element IRS with quantized BCD method, target rate of 6bits/s/Hz and ξ=20.

In Figure 8, the spatial distributions of UEs that satisfy the target rate are illustrated. The target rates are set to 7 and 6bits/s/Hz for ξ=1 (Figure 8a–c) and ξ=20 (Figure 8d–f), respectively. Note that, by observing uniformly distributed 4000 UEs within the considered area, the increased number of UEs who satisfy the target rate can be interpreted as the growth of the coverage area. When ξ=1, only 9.43% of the UEs were satisfied the target rate of 7bits/s/Hz, while 59.95% and 69.93% of the UEs were satisfied, for IRS with BCD and quantized BCD methods, respectively. Compared to the IRS with the BCD method, about 10% of coverage is extended by adopting the quantized BCD method. Similarly, in Figure 8e, 20.98% of coverage is obtained by utilizing the analog BCD method, which is even lower than the system without IRS. However, with the quantized BCD method in Figure 8f, approximately 20% larger coverage is obtained compared to the system without IRS, which can also be verified in Figure 7.

## 6. Conclusions

In this study, the IRS discrete phase shift method is investigated to reduce the network overhead of IRS-aided multiple-antenna systems. Finding the optimal resolution of the discrete phase shift that maximizes the overhead-aware achievable rate is a combinatorial problem demanding substantial computational complexity. Considering the concavity of the achievable rate model, the proposed incremental search-based method with two low-complexity sub-algorithms can efficiently find the optimal resolution. Furthermore, it is numerically shown that the superiorities between two sub-algorithms exist under the various communication parameters. Consequently, the proposed method can improve by more than 16% of the achievable rate compared to the existing analog phase shift method when the 1024-element IRS is adopted. Moreover, about 20% of the coverage can be extended by using the proposed method with 576-element IRS.

## Figures and Tables

**Table 1:** Simulation Environment Parameters [81].

Parameters	Values
Coverage area	$500\times 250\;m^{2}$
BS/IRS locations	(0,0,10)/(250,250,5)m
UE location	(x,y,0)m, where x∈[100,400] and y∈[50,250]
Azimuth angles for BS and UE	$\theta_{\mathrm{AoD},B}=0.7853$, $\theta_{\mathrm{AoA},U}=-0.3216$
Azimuth angles for IRS	$\theta_{\mathrm{AoA},R}=3.9270$, $\theta_{\mathrm{AoD},R}=1.8925$
Elevation angles for IRS	$\phi_{\mathrm{AoA},R}=0.0141$, $\phi_{\mathrm{AoD},R}=0.0316$
Number of IRS elements	N∈{16,64,144,256,400,576,784,1024}
Bandwidth/carrier frequency [83]	W=10MHz / $f_{c}=2.5\;\mathrm{GHz}$
Antenna (IRS element) spacing [82]	Half wavelength, i.e., $d=\lambda_{W}/2=0.0075\;m$
Downlink duration/pilot overhead parameter	T=10msec / ξ∈[1,40]
Rician factor [58]	$K_{f}=K_{g}=10\;\mathrm{dB}$
Noise Figure [58]	−174dBm/Hz
Antenna gain for BS/UE [58]	$G_{B}=5\;\mathrm{dBi}$/$G_{U}=5\;\mathrm{dBi}$
Pathloss for Rician [83]	$\eta \left(d_{a,b}\right)=G_{B}+G_{U}-28-20\log_{10}\left(f_{c}\right)-22\log_{10}\left(d_{a,b}\right)$
Pathloss for Rayleigh [83]	$\eta \left(d_{a,b}\right)=G_{B}+G_{U}-22.7-26\log_{10}\left(f_{c}\right)-36.7\log_{10}\left(d_{a,b}\right)$
Computer/simulator	3.0-GHz CPU and 32-GB RAM / MATLAB-2021a

## Abbreviations

The following abbreviations are used in this manuscript:

AoA	Angle of arrival
AoD	Angle of departure
BCD	Block coordinate descent
BS	Base station
CSI	Channel state information
IRS	Intelligent reflecting surface
LoS	Line of sight
MISO	Multiple-input multiple-output
MRT	Maximum ratio transmission
NLoS	Non-line-of-sight
NOMA	Non-orthogonal multiple access
PIN	Positive–intrinsic–negative
SNR	Signal-to-noise ratio
STBC	Space–time block code
STLC	Space–time line code
UCR	Unit-modulus constraint
UE	User equipment
